# Time course of neuroinflammation after human stroke – a pilot study using co-registered PET and MRI

**DOI:** 10.1186/s12883-023-03178-7

**Published:** 2023-05-16

**Authors:** Lucio D’Anna, Graham Searle, Kirsten Harvey, Paul M. Matthews, Roland Veltkamp

**Affiliations:** 1 Department of Stroke and Neuroscience, Charing Cross Hospital, Imperial College Healthcare NHS Trust, London, UK; 2 Department of Brain Sciences, Imperial College London, London, UK; 3 Invicro, A Konica Minolta Company, London, UK; 4 Dementia Research Institute at Imperial College London, London, UK; 5 Department of Neurology, Alfried-Krupp Krankenhaus Essen, Essen, Germany; 6 Department of Neurology, University Hospital Heidelberg, Heidelberg, Germany

**Keywords:** Neuroinflammation, Microglia, Benzodiazepine receptor, Brain ischemia

## Abstract

**Background:**

Microglial activation contributes to both inflammatory damage and repair in experimental ischemic stroke. However, because of the logistical challenges, there have been few clinical imaging studies directly describing inflammatory activation and its resolution after stroke. The purpose of our pilot study was to describe the spatio-temporal profile of brain inflammation after stroke using 18kD translocator protein (TSPO) positron emission tomography (PET) with magnetic resonance (MR) co-registration in the subacute and chronic stage after stroke.

**Methods:**

Three patients underwent magnetic resonance imaging (MRI) and PET scans with TSPO ligand [^11^C]PBR28 15 ± 3 and 90 ± 7 days after an ischaemic stroke. Regions of interest (ROI) were defined on MRI images and applied to the dynamic PET data to derive regional time-activity curves. Regional uptake was quantified as standardised uptake values (SUV) over 60 to 90 min post-injection. ROI analysis was applied to identify binding in the infarct, and in frontal, temporal, parietal, and occipital lobes and cerebellum excluding the infarcted area.

**Results:**

The mean age of participants was 56 ± 20.4 years and mean infarct volume was 17.9 ± 18.1 ml. [^11^C]PBR28 showed increased tracer signal in the infarcted area compared to non-infarcted areas of the brain in the subacute phase of stroke (Patient 1 SUV 1.81; Patient 2 SUV 1.15; Patient 3 SUV 1.64). [^11^C]PBR28 uptake returned to the level of non-infarcted areas at 90 days Patient 1 SUV 0.99; Patient 3 SUV 0.80). No additional upregulation was detected elsewhere at either time point.

**Conclusions:**

The neuroinflammatory reaction after ischaemic stroke is limited in time and circumscribed in space suggesting that post-ischaemic inflammation is tightly controlled but regulatory mechanisms.

**Supplementary Information:**

The online version contains supplementary material available at 10.1186/s12883-023-03178-7.

## Background

There is a substantial unmet medical need for therapies that improve outcome after ischaemic stroke. Experimental studies suggest that inflammatory processes contribute to secondary injury after brain ischaemia [1, 2]. Brain injury following transient or permanent focal cerebral ischaemia develops from a complex series of pathophysiological events that evolve in time and space [3]. Focal cerebral ischaemia induces a time-dependent recruitment and activation of inflammatory cells, including neutrophils, T cells, and monocytes/macrophages [4, 5].

The microglia are activated within hours after ischaemia-onset and secrete proinflammatory cytokines, which increase brain damage. Subsequently, microglia produce anti-inflammatory and neurotrophic factors that promote regeneration and plasticity [6, 7]. The translation of promising preclinical studies addressing neuroinflammatory targets into the clinical setting has been unsuccessful so far [7]. One major limitation to their translation has been the limited understanding of the timing of inflammatory mechanisms and their anatomic distribution in the human brain after stroke.

Positron emission tomography (PET) radiotracers have been developed as noninvasive molecular imaging tools to assess neuroinflammation [8]. Translocator protein 18 kDa (TSPO), previously known as the peripheral benzodiazepine receptor, is present mainly in the mitochondrial compartment and can be imaged in vivo using PET [9]. TSPO is overexpressed by microglia and monocyte-derived cells during inflammation and has been extensively used as a measure of inflammation in numerous neurological diseases including Alzheimer’s disease, Parkinson’s disease and multiple sclerosis [9–12].

The first non-benzodiazepine-type compound to be radiolabeled was PK11195 ([^11^C]PK-11195) [13]. As a selective radioligand of TSPO it has been used to study the magnitude of inflammation after acute ischaemic stroke in patients [14–18] but several methodological issues have limited the usefulness of this radioligand [8]. Subsequently, second generation TSPO radiotracers with more favourable pharmacokinetic properties, including [^11^C]PBR28, have been developed. This has resulted in a substantially improved signal-to-noise ratio [19]. Thus far, only a small number of studies have examined TSPO tracer binding in co-registration with MRI-based structural imaging data to depict the anatomical distribution of tracer binding relative to the infarct [17, 18]. Moreover, insights into the temporal course of tracer binding are limited.

The purpose of our pilot study was to describe the activation of microglia by in vivo brain expression of TSPO using [^11^C]PBR28 PET imaging co-registered with MRI structural imaging in the subacute and chronic stages after ischaemic stroke.

## Methods

Patients with clinically and radiologically defined supratentorial ischaemic stroke within the previous 10 days were studied sequentially at 15 ± 3 and 90 ± 7 days after the event. To be included, patients had to: understand the purpose and risks of the study; provide informed consent; and give authorization for the use of their protected health information. These processes were carried out in accordance with national and local subject privacy regulations and following local institutional review board’s (IRB’s)/ethics committee’s (EC’s) guidelines. The severity of neurological signs was rated according to the National Institutes of Health Stroke Scale (NIHSS). The Modified Rankin Scale (mRS) was used to assess the level of functional independence. Key exclusion criteria included the presence of acute intracranial haemorrhage on acute brain imaging; contraindications for undergoing MRI examination (including metal foreign bodies, cardiac pacemakers, renal impairment that contraindicates gadolinium) or PET scan; females who were pregnant, nursing, or planning to become pregnant during study participation; patients carrying genetic polymorphism consistent with low TSPO binding affinity. Additionally, patients were excluded if they had participated in a research or medical protocol in the previous 12 months which involved nuclear medicine, PET or radiological investigations with radiation exposure that, when combined with the radiation exposure from the present study, would exceed 10 mSV in addition to the natural background radiation. All scans were performed at the Invicro-London clinical imaging centre at Hammersmith Hospital Campus, Imperial College London.

### PET procedure

[^11^C]-PBR28 was prepared by reacting [^11^C]-methyl iodide with PBR28 precursor, 4-[N-Acetyl-N-[2-methoxybenzyl]amino]-3-phenoxypyridine. The Good Manufacturing Practice (GMP) grade precursor was supplied by Pharmasynth with specification set to “ > 95%” for purity (as measured by HPLC). [^11^C]-PBR28 was formulated in 11 mL of maximum 9% (v/v) ethanol in 0.9% saline solution for injection. The automated synthetic procedure of [^11^C]-PBR28 was developed in-house using an Eckert and Ziegler Modular Lab system coupled with a semi-preparative HPLC system. The product was radiochemical pure before all injections.

Subjects were positioned in the PET scanner after the insertion of a venous cannula in an antecubital or forearm vein. A head-fixation device was used to minimise head movement during data acquisition. All dynamic [^11^C]-PBR28 PET scans were acquired on Siemens PET/CT scanners; either a Biograph 6 or a Biograph 6 TruePoint with TrueV PET/CT scanner (Siemens Healthcare, Erlangen, Germany). A low-dose CT scan was performed immediately before each PET study to estimate attenuation. Following intravenous bolus injection of the radiotracer, dynamic emission data were acquired for 90 min (frame durations: 8 × 15 s, 3 × 60 s, 5 × 120 s, 5 × 300 s, 5 × 600 s) for all scans except one (which was aborted at 79 min at the subject’s request). The dynamic images were reconstructed using Fourier rebinning and a 2D filtered discrete inverse Fourier transform algorithm with 5 mm isotropic Gaussian filter on a 128 × 128 matrix with 2.6 zoom giving 2 mm isotropic voxels. Corrections were applied for attenuation, randoms and scatter.

### MRI procedure

MRI acquisitions were carried out on a Siemens 3 T MRI scanner (Siemens Healthineers, Erlangen, Germany). To assess the size and location of the infarct, diffusion-weighted images (DWI, using a b-factor of 1,000 s/mm^2^) and T2-FLAIR-weighted images (repetition time 5000 ms; echo time 387 ms; 192 slices) were acquired. T1-weighted magnetization-prepared rapid gradient echo (MPRAGE) sequences were acquired with the following parameters: repetition time 2,300 ms; echo time 2.98 ms; flip angle, 9; 160 slices. Each study participant received 0.2 ml/kg dose of gadolinium (Dotarem 0.5 mmol/ml solution).

### Image analysis

The location and extent of the index brain infarcts were initially identified on routine clinical DWI MR images. The clinical MR images (T1, DWI and T2 FLAIR) performed during the hospital admission were inspected and compared with the research scans performed to exclude any stroke recurrence in between. The ischaemic infarct was delineated manually using MRICroN software (version 12/2009; C. Rorden) [20] in native space to obtain the volume taking into account the location and extent of the region(s) identified in the original DWI, and FLAIR images. Pre- and post-gadolinium T1-weighted images were compared in order to assess enhancement.

T1 MPRAGE images underwent grey matter segmentation and were co-registered to a standard reference space (MNI152;) [21]. The MNI52 template brain image and associate atlas (CIC atlas) was nonlinearly warped to the subject’s MR image to enable automated definition of other regions of interest (ROIs), including frontal lobe, temporal lobe, parietal lobe, occipital lobe and cerebellum in the left and right hemisphere and bilateral ROIs merged together with the exclusion of the territory involved in the infarct. The manually drawn ischemic infarct ROI was aligned with the subject’s T1 MPRAGE via registration of the T2 FLAIR to the T1 MPRAGE image. The aligned infarct ROI image was applied as a mask to remove the infarct region from the atlas-derived regions (such as frontal lobe).

Dynamic PET images were registered to each subject’s MRI scan and corrected for motion using a frame-to-frame registration process with a normalised mutual information cost function. ROIs defined on the MRI images were applied to the dynamic PET data to derive regional time-activity curves (TACs). Regional uptake was quantified as standardised uptake values (SUV) over the period from 60–90 min post-injection. SUV ratio (SUVR) values were also calculated, using cerebellum as the reference.

## Results

Three patients (2 female, 1 male) with a supratentorial acute ischemic stroke were included in the study (Supplemental Table 1). The mean age of the patients was 56 ± 20.4 years. The mean NIHSS score on original admission was 9 ± 4.6. The mean infarct volume was 17.9 ± 18.1 ml. None of the patients had clinical signs of infection or raised inflammatory parameters during the acute stage, and on the days of baseline and follow-up images.

**Fig. 1:**
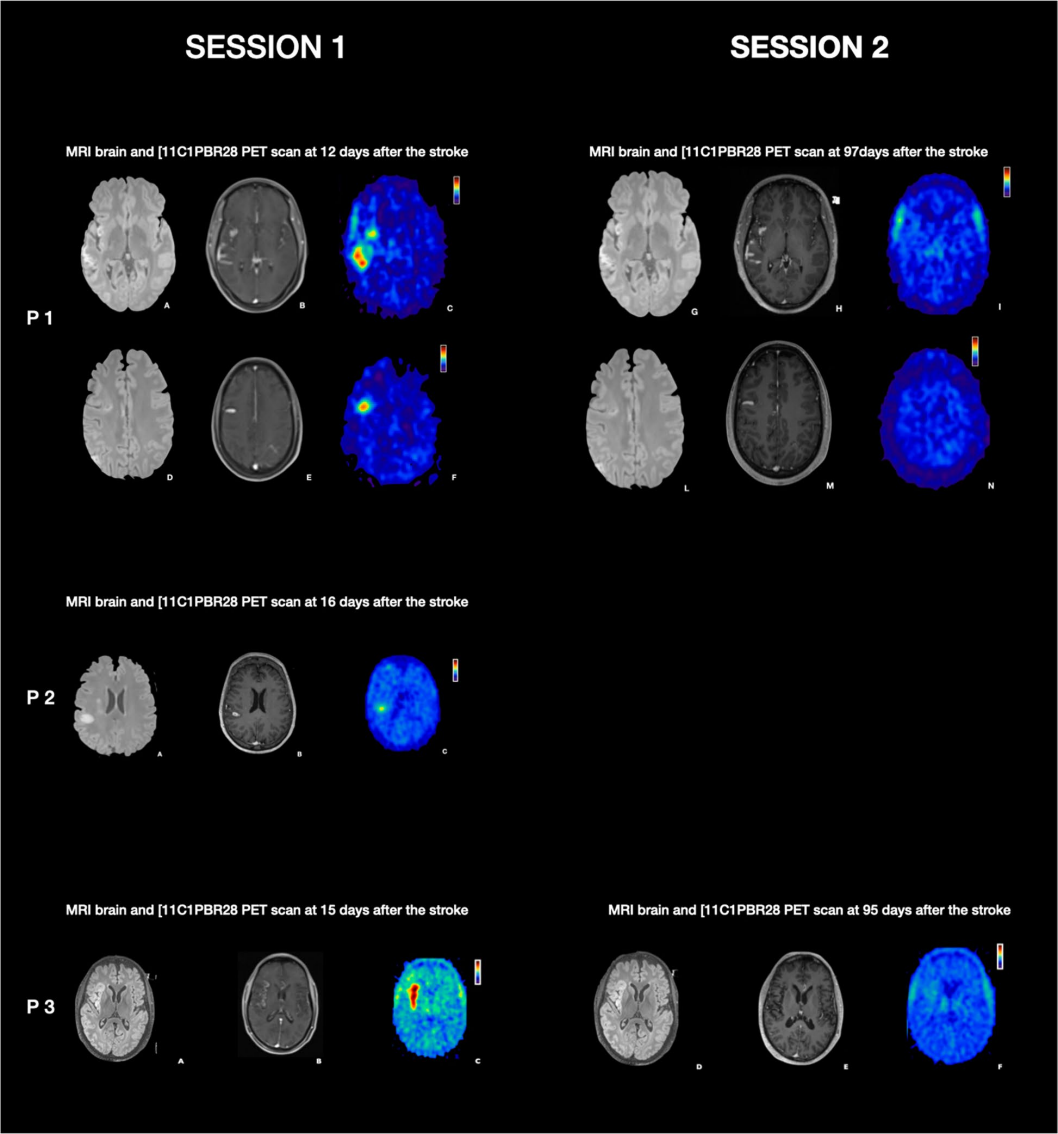
P1. The T2 FLAIR and the post contrast T1 MPRAGE images (**A**, **B**, **D**, **E**) showed right middle cerebral artery and anterior cerebral artery infarcts. The [^11^C]PBR28 PET scan illustrated an increased PBR28 signal corresponding to the core stroke areas (**C**, **F**) at 12 days after the stroke. At 97 days after the stroke the [^11^C]PBR28 PET scan showed that the PBR28 signal in the infarcted areas was disappeared (**I**, **N**). P2. The T2 FLAIR and the post contrast T1 MPRAGE images showed the stroke core area (**A**, **B**) and the [^11^C]PBR28 PET scan showed an increased PBR28 signal corresponding to the core stroke area at 16 days after the stroke (**C**). P3. The T2 FLAIR and the post contrast T1 MPRAGE images (**A**, **B**) the stroke core area. The [.^11^C]PBR28 PET scan showed an increased PBR28 signal (**C**) corresponding to the core stroke area identified. At 95 days following the stroke the PBR28 signal in the infarcted area disappeared **(F**)

Patient 1 had been treated with mechanical thrombectomy because of a right M1 occlusion resulting in right middle cerebral artery infarcts. The first scanning session was performed twelve days after the stroke when her NIHSS was 1 (residual mild dysarthria) and her mRS was 1. The [^11^C]PBR28 PET scan of the patient revealed an increased PBR28 signal corresponding to the infarcted area on T2 FLAIR images (Fig. 1). Post-contrast T1w imaging revealed enhancement in a part of the infarct. The SUV and SUVR values were increased in the infarcted area compared to the ROIs that included the frontal lobe, temporal lobe, parietal lobe, occipital lobe and cerebellum in the left and right hemisphere and compared to the bilateral ROIs merged together with the exclusion of the territory involved in the infarct (Table 1) (Supplemental Table 2 and 3). The second scanning session was performed at ninety-seven days following the stroke when NIHSS and mRS scores remained unchanged. The increased PBR28 signal was no longer visible in the infarcted areas in the second [^11^C]PBR28 PET scan (Fig. 1); the SUV and SUVR values of the infarcted areas were similar to those in other predefined ROIs remote from the stroke (Table 1) (Supplemental Tables 2 and 3).

**Table 1:** Regional Standardized Uptake Value ratio (SUVR) values in the ischemic core and in other Region of Interests (ROIs) at session 1 and session 2

Patient Session	Ischaemic core	Bilateral Frontal lobe (grey matter-no-infarcted area)	Bilateral Temporal lobe (grey matter-no-infarcted area)	Bilateral Parietal lobe (grey matter-no-infarcted area)	Bilateral Occipital lobe (grey matter-no-infarcted area)	Bilateral Cerebellum (grey matter-no-infarcted area)
P1/S1	1.81	0.92	1.00	0.93	0.91	1.00
P2/S1	1.31	1.01	1.03	1	1.01	1.00
P3/S1	2.38	1.13	1.00	1.00	0.96	1.00
P1/S2	1.01	0.89	0.92	0.94	0.91	1.00
P3/S2	1.23	1.00	1.01	0.92	0.95	1.00

*P* Patient, *S* Session

Patient 2 experienced a right middle cerebral artery infarct predominantly in the territory of the peripheral lenticular striate vessels. The first scanning session was performed sixteen days after the stroke, when her NIHSS was 2 (left minor facial palsy and drift of her left leg) and her mRS was 1. The [^11^C]PBR28 PET scan showed an increased PBR28 signal corresponding to the infarcted area as identified on T2 FLAIR (Fig. 1). The SUV and SUVR values corresponding to the infarct were increased when compared to the predefined ROIs (Table 1) (Supplemental Tables 2 and 3). Patient 2 subsequently declined undergoing the second scanning session.

Patient 3 suffered an acute occlusion of the M1 segment of the right middle cerebral artery for which he was treated with intravenous thrombolysis and mechanical thrombectomy. The first scanning session was performed fifteen days after the stroke, when his NIHSS was 0 and his mRS was 0. The PET scan visibly demonstrated an increased [^11^C]PBR28 uptake (Fig. 1), also measured by SUV and SUVR in the infarct areas compared to the predefined ROIs (Table 1) (Supplementals Table 2 and 3). The observed increased PBR28 signal corresponded to the infarct as identified on T2 FLAIR images (Fig. 1).

The second scanning session was performed 95 days following the stroke. His NIHSS was unchanged, but his mRS was 1. Elevated PBR28 signal in the infarcted areas was no longer visible (Fig. 1). The [^11^C]PBR28 uptake measured by SUV and SUVR in the infarcted area returned to values observed in other predefined ROIs outside the infarct (Table 1) (Supplemental Tables 2 and 3).

## Discussion

The key findings of our pilot study are that the TSPO ligand [^11^C]PBR28 showed a transient increased signal, only within the infarcted area, in the subacute phase of stroke and that this evidence of circumscribed glial activation resolved within 90 days.

Previous TSPO PET studies documented an increased early uptake of PK11195, a first generation TSPO ligand, in the infarct zone and suggested increases in binding up to 30 days after stroke [14, 15, 22]. Using the second generation TSPO tracer [^11^C]vinpocetine, Gulyás and colleagues measured the regional changes of TSPO in the brain of nine ischemic stroke patients up to 14 weeks after the insult. The authors reported increased radioligand uptake in both the ischaemic core and the peri-infarct zone [22].

The radioligand [^11^C]PBR28 has higher specific and lower nonspecific binding to TSPO than the first generation radioligands [8]. Hence, it is capable of assessing the local accumulation of CNS resident inflammatory cells (primarily microglial and astrocytes) with high sensitivity and specificity and to localise inflammation relatively precisely. With preclinical PET, its use for mapping the inflammatory response to relatively small areas such as the peri-ischemic penumbra in a rat stroke model, was validated [23]. In a single case study, Kreisl and colleagues described an unusual occurrence of stroke presenting as an incidental finding of increased binding of [^11^C]PBR28 in the right basal ganglia on PET imaging [24–26]. The patient was a 42-year-old man that subsequently recalled a previously unreported episode of mild weakness in his left face and left hand that occurred 12 days before the PET scan and had resolved completely within twenty-four hours. His brain MRI showed only occasional foci of mild chronic white matter ischemia but was otherwise unremarkable.

Our study is the first to sequentially study the pattern of [^11^C]PBR28 over a period of 90 days in patients with acute ischaemic stroke. Our findings provide scientific rationale to support that TSPO imaging provides a valuable tool to study the neuroinflammatory response in stroke and allows for assessment of immunomodulatory treatments targeting inflammation in pre-clinical and clinical settings. The possibility that increased [^11^C]PBR28 signal within the infarct borders is a result of increased post-ischaemic blood–brain barrier permeability is contradicted by the observation that post-contrast enhancement on T1w MR images persisted in patient 3 at 90 days whereas increased tracer signal had subsided. Moreover, previous studies revealed that the degree of microglial activation appeared to be directly correlated with the degree of white matter fibre damage on MRI, which persisted in longitudinal TSPO PET assessments for up to six months after the index stroke [27, 28]. Hence we conclude that the elevated [^11^C]PBR28 signal observed in this study is likely to result primarily from binding of TSPO rather than distribution effects.

The biological effects of the local microglial/macrophage and astrocyte recruitment after stroke [25, 26], which our data reflect, are complex and time-dependent. Different subsets of microglia fulfil multiple roles after ischaemic stroke [19]. In the early phase of experimental ischemic stroke, microglia produce pro-inflammatory mediators such as TNF-α, IL-1β and reactive oxygen species resulting in further damage [6, 29] and secondary cell death in the penumbra region [30]. However, microglia also secrete anti-inflammatory factors after ischemic stroke [31, 32] which may promote neurogenesis and plasticity [6, 33]. In the context of an acute injury, appropriate astrocyte-microglia cross-talk is necessary for astrocytes to support neuronal survival and function [33]. An important finding of our study is that focal inflammation after stroke in patients is self-limiting in time and circumscribed in space. The localisation of the inflammatory response, within the borders of the infarct in the subacute phase, suggests that wide-spread activation of the resident cerebral immune cells is prevented by intrinsic mechanisms of inflammatory control [34]. Moreover, even within the infarct, our findings suggest that the accumulation of inflammatory cells resolves within 3 months after the event.

Our pilot study has limitations. The very stringent exclusion criteria resulted in a small sample size, therefore, our findings have to be confirmed in larger studies. Moreover, we did not collect arterial blood, but used SUV_60-90_ as the primary outcome measure. The SUV could subject to confounds with differences in peripheral TSPO binding (e.g., to lymphocytes) that would reduce the free radioligand concentration with significant systemic inflammation [35]. Previous studies have documented a strong regional overlap between [^11^C]PBR28 SUV_60-90_ and distribution volume [36, 37]. Finally, our 3 patients differed considerably in terms of age and in terms of vascular territory affected by the stroke.

## Conclusions

TSPO PET imaging during the subacute phase suggests that the inflammatory response is confined to the infarcted area in stroke patients, which then resolves within three months. This also establishes a limited time window for administration of therapeutic anti-inflammatory agents, and could emphasise that locally delivered treatments could be effective. Further insights into the neuroinflammatory response after stroke are needed to guide the development of therapies targeting harmful and beneficial aspects of post-stroke inflammation.

## Supplementary Information

**Additional file 1: Supplemental Table 1.** Patient Clinical Summary including Age, Vascular Territory, NIHSS on admission, and Time points of combined MRI and PET scans. **Supplemental Table 2.** Regional Standardized Uptake Value (SUV) values in different brain regions at session 1 and session 2. **Supplemental Table 3.** Regional Standardized Uptake Value (SUV) values in different brain regions at session 2. **Supplemental Table 4.** Patient data including specific activity and mass of [11C]PBR28 administered and Standardized Uptake Value (SUV) time.

## Data Availability

Data and materials available upon reasonable request.

## Abbreviations

TSPO: 18KD Translocator protein PET
MR: Magnetic resonance
SUV: Standardised uptake values
ROI: Regions of interest
PET: Positron emission tomography
NIHSS: National Institutes of Health Stroke Scale
mRS: Modified Rankin Scale
TAC: Time-activity curves
DWI: Diffusion Weighted Images
