# Supplementary material for: Time course of neuroinflammation after human stroke – a pilot study using co-registered PET and MRI
Source: BMC Neurol. 2023 May 16;23:193. doi: 10.1186/s12883-023-03178-7 (PMC10186673; doi:10.1186/s12883-023-03178-7)
Supplement: Supplementary file 1 — Additional file 1: Supplemental Table 1. Patient Clinical Summary including Age, Vascular Territory, NIHSS on admission, and Time points of combined MRI and PET scans. Supplemental Table 2. Regional Standardized Uptake Value (SUV) values in different brain regions at session 1 and session 2. Supplemental Table 3. Regional Standardized Uptake Value (SUV) values in different brain regions at session 2. Supplemental Table 4. Patient data including specific activity and mass of [11C]PBR28 administered and Standardized Uptake Value (SUV) time. [file 12883_2023_3178_MOESM1_ESM.docx]

**Supplemental Table 1. Patient Clinical Summary including Age, Vascular Territory, NIHSS on admission, and Time points of combined MRI and PET scans.**

| **Patient no./sex** | **Age (year)** | **Admission NIHSS score** | **Vascular territory** | **Infarct volume (mL)** | **Combined PET and MRI image Time, d** |
| --- | --- | --- | --- | --- | --- |
| P1/Female | 33 | 8 | R MCA and ACA | 14.44 | ± 15, ± 90 |
| P2/Female | 72 | 5 | L MCA | 1.80 | ± 15 |
| P3/Male | 63 | 14 | R MCA | 37.47 | ± 15, ± 90 |

NIHSS: National Institutes of Health Stroke Scale; Patient: P; L left; R right; MCA middle cerebral artery.

**Supplemental Table 2. Regional Standardized Uptake Value (SUV) values in different brain regions at session 1 and session 2.**

Patient: P; Session: S.

| **Patient Session** | **Ischemic core** | **Left Frontal lobe (grey matter-no-infarcted area)** | **Left Temporal lobe**  **(grey matter-no-infarcted area)** | **Left Parietal lobe**  **(grey matter-no-infarcted area)** | **Left Occipital lobe**  **(grey matter-no-infarcted area)** | **Left Cerebellum**  **(grey matter-no-infarcted area)** | **Right Frontal lobe (grey matter-no-infarcted area)** | **Right Temporal lobe**  **(grey matter-no-infarcted area)** | **Right Parietal lobe**  **(grey matter-no-infarcted area)** | **Right Occipital lobe**  **(grey matter-no-infarcted area)** | **Right**  **Cerebellum**  **(grey matter-no-infarcted area)** |
| --- | --- | --- | --- | --- | --- | --- | --- | --- | --- | --- | --- |
| P1/S1 | 1.81 | 0.86 | 0.88 | 0.93 | 0.91 | 1.00 | 0.99 | 1.13 | 0.92 | 0.92 | 1.00 |
| P2/S1 | 1.15 | 0.87 | 0.91 | 0.88 | 0.89 | 0.88 | 0.90 | 0.93 | 0.91 | 0.90 | 0.88 |
| P3/S1 | 1.64 | 0.74 | 0.69 | 0.69 | 0.66 | 0.69 | 0.81 | 0.71 | 0.70 | 0.67 | 0.72 |

| **Patient Session** | **Ischaemic core** | **Bilateral Frontal lobe**  **(grey matter-no-infarcted area)** | **Bilateral Temporal lobe**  **(grey matter-no-infarcted area)** | **Bilateral Parietal lobe**  **(grey matter-no-infarcted area)** | **Bilateral Occipital lobe**  **(grey matter-no-infarcted area)** | **Bilateral Cerebellum**  **(grey matter-no-infarcted area)** |
| --- | --- | --- | --- | --- | --- | --- |
| P1/S2 | 0.99 | 0.87 | 0.90 | 0.92 | 0.89 | 0.98 |
| P3/S2 | 0.80 | 0.65 | 0.66 | 0.60 | 0.62 | 0.65 |

Patient: P; Session: S.

**Supplemental Table 3. Regional Standardized Uptake Value (SUV) values in different brain regions at session 2.**

| **Patient Session** | **Ischemic core** | **Left Frontal lobe**  **(grey matter-no-infarcted area)** | **Left Temporal lobe**  **(grey matter-no-infarcted area)** | **Left Parietal lobe**  **(grey matter-no-infarcted area)** | **Left Occipital lobe**  **(grey matter-no-infarcted area)** | **Left Cerebellum**  **(grey matter-no-infarcted area)** | **Right Frontal lobe**  **(grey matter-no-infarcted area)** | **Right Temporal lobe**  **(grey matter-no-infarcted area)** | **Right Parietal lobe**  **(grey matter-no-infarcted area)** | **Right Occipital lobe**  **(grey matter-no-infarcted area)** | **Right Cerebellum**  **(grey matter-no-infarcted area)** |
| --- | --- | --- | --- | --- | --- | --- | --- | --- | --- | --- | --- |
| P1/S2 | 0.99 | 0.86 | 0.87 | 0.92 | 0.88 | 0.98 | 0.88 | 0.92 | 0.91 | 0.90 | 0.99 |
| P3/S2 | 0.80 | 0.63 | 0.65 | 0.60 | 0.62 | 0.66 | 0.68 | 0.70 | 0.61 | 0.62 | 0.64 |

Patient: P; Session: S.

**Supplemental Table 4. Patient data including specific activity and mass of [^11^C]PBR28 administered and Standardized Uptake Value (SUV) time.**

| **Patient Session** | **Injected Activity (MBq)** | **Injected mass (ug)** | **SUV time (min)** |
| --- | --- | --- | --- |
| P1/S1 | 133 | 1.62 | 60-79 |
| P1/S2 | 239 | 3.49 | 60-90 |
| P2/S1 | 233 | 3.66 | 60-90 |
| P3/S1 | 189 | 3.05 | 60-90 |
| P3/S2 | 256 | 3.49 | 60-90 |

Patient: P; Session: S.
